# Editorial: Unawareness of Illness in Neurological Disorders: A Focussed Neurocognitive Approach Shedding Light on Neuropsychological Deficits and Neural Underpinnings Potential Association

**DOI:** 10.3389/fpsyg.2020.622576

**Published:** 2020-12-07

**Authors:** Martina Amanzio, Sara Palermo

**Affiliations:** ^1^Department of Psychology, University of Turin, Turin, Italy; ^2^Department of Neuroscience, Center for the Study of Movement Disorders, University of Turin, Turin, Italy; ^3^European Innovation Partnership on Active and Healthy Aging, Brussels, Belgium

**Keywords:** illness unawareness, assessment, theoretical models, executive dysfunction, neurodegenerative and vascular brain injuries, neuroimaging, electrophysiological techniques

## Introduction

Awareness of disease is a multidimensional construct, defined in different terms and with reference to specific theories. Moreover, it can be observed in subjects suffering from neurodegenerative, vascular diseases, and psychiatric disorders.

The study of disease awareness can be divided into at least three main components: (1) a lack of awareness of symptoms; (2) a lack of awareness of the consequences of a disorder, and (3) a lack of awareness of the need to be treated. Explanatory definitions of reduced awareness of a disease presented in the literature are different and can be associated with specific conceptual reference models.

This type of clinical disorder can be defined differently depending on the type of disease from which the patient suffers. Reduced awareness of different types of psychiatric and neurological illnesses is defined using different terms:

1. *Anosognosia* typically describes a failure, often observed in neurological patients, to acknowledge a particular neuropsychological deficit concerning specific modular functions (perception, action, or language).
2. *Lack of insight* usually describes a lack of introspective knowledge in psychiatric illness. In psychiatry, another term used is *denial*, which is a defense mechanism in which the existence of unpleasant internal or external realities is denied and kept out of consciousness.
3. *Awareness of illness* is referred to as a form of knowledge derived from the ability of patients to recognize their disturbances and errors. A reduction in self-awareness related to executive dysfunction is well-described in different disorders, from neurodegenerative to vascular pathologies.

Studies using the neurocognitive approach, which analyses a reduction in awareness by integrating neurobiological and neuropsychological levels of explanation (in terms of brain dysfunction and concomitant cognitive-behavioral disturbance) may have greater clinical relevance. Indeed, clinicians and patients can benefit from integrating this perspective into their practice, from diagnosis and prognosis to cognitive-functional improvement and rehabilitation treatment (Amanzio et al., 2011; Palermo et al., 2014).

This editorial is a brief overview of contributions to this Research Topic. We encourage a thorough reading of the articles for a complete understanding of “awareness of illness” in neurological disorders and neuropsychiatric patients.

This Research Topic collects seven contributions, including three review papers, two original research articles, and two case reports.

## Theoretical Issues

Illness unawareness is a common feature of major neurocognitive disorders, such as Alzheimer's Disease (Lenzoni et al.).

It has been hypothesized that memory disorder may be a key contributing factor, as patients are unable to update information about their performance and rely on what has been previously experienced in terms of their past functional ecological performance and neuropsychological testing. This characterizes what has been termed “the petrified self” (Mograbi et al., 2009).

In their review, Lenzoni et al. presented evidence from the past 10 years and focused on anterograde memory deficits about performance, the profile of autobiographical retrograde memory loss, and the role of frontal lobes.

A divergent line of investigation has shown that illness unawareness can be fruitfully explained by considering both prefrontal cortex anatomo-functional changes and executive dysfunction in patients with Alzheimer's Disease, behavioral frontotemporal dementia, acquired brain injury, and Parkinson's Disease (Amanzio et al., 2011, 2013, 2014, 2016, 2017, 2018; Palermo et al., 2014, 2017, 2018). In line with these findings, a reduction in self-awareness could be explained by considering the executive dysfunction associated with prefrontal cortex anatomo-functional changes (Amanzio et al.).

The authors highlight the results obtained through a neurocognitive approach combined with a theoretical model. The data suggest that the key role of executive functions and mood deflections in sustaining adequate self-awareness in the instrumental activity of daily living (Amanzio et al.).

## Unawareness in Acquired Brain Injury

Awareness is frequently impaired after acquired brain injury and may reduce a subject's compliance with treatment, worse functional outcome, and as a result, high caregiver distress (Bivona et al.). Considering the multifaceted nature of self-awareness, a specific and effective assessment is crucial. In their study, Bivona et al. proposed the use of the Self-Awareness Multilevel Assessment Scale (SAMAS). Findings showed that SAMAS can provide an accurate diagnosis of illness unawareness, thus better addressing neurorehabilitation treatment and, accordingly, reducing the possible occurrence of its primary and secondary implications (Bivona et al.).

Selective vascular lesions offer an opportunity to investigate the key neuropsychological features of illness unawareness in neurocognitive disorders. Because of its rarity, a case report presented an unusual case of a woman affected by a combined polar and paramedian bilateral thalamic infarction (Bartoli et al.). The patient developed executive dysfunction associated with reduced self-awareness and mood changes, in terms of apathy and depression. Importantly, the patient underwent a serology test in chemiluminescence to detect IgG antibodies against SARS-CoV-2. Authors suggested that metacognitive–executive dysfunction, which can affect autonomy in instrumental abilities, might make people less able to take appropriate precautions, facilitating the risk of SARS-CoV-2 contagion (Bartoli et al.).

## Unawareness and Denial in Traumatic Brain Injury and Schizoaffective Disorder

If we consider literature from the last 20 years on traumatic brain injury, the role of denial in illness unawareness has typically not been addressed. For the first time, a review article proposed early findings in the field and integrated those findings with more recent observations (Prigatano and Sherer). The authors suggest that this synthesis of information and expert clinical opinion will inform future research on impaired self-awareness and denial as well as approaches to rehabilitation for persons with traumatic brain injury (Prigatano and Sherer).

Denial was also considered in the case report presented by Prigatano et al.. Persistent denial of severe and acute pain following orthopedic injuries has not been previously reported. The authors describe a case of a 24-year-old woman with a history of schizoaffective disorder who suffered severe pain secondary to acute orthopedic injuries. A modified version of the Conscious Avoidance subscale of the Denial of Illness Questionnaire was useful in measuring the severity level of her denial (Prigatano et al.). Authors suggested that the behavioral features of psychological denial appear different from those associated with impaired self-awareness secondary to an underlying brain disorder (Prigatano et al.).

## Self-Awareness in Cervical Dystonia

Cervical dystonia is a focal disorder characterized by anomalies of sensorimotor integration and proprioceptive dysfunctions, which can have an impact on body awareness. Innovative research by Ferrazzano et al. assessed whether patients may have a compromised awareness of dystonic posture. Contrary to the hypothesis of the authors, most patients with this specific disorder seem to have a preserved awareness of their dystonia and tremors (Ferrazzano et al.).

## Author Contributions

All authors listed have made a substantial, direct and intellectual contribution to the work, and approved it for publication.

## Conflict of Interest

The authors declare that the research was conducted in the absence of any commercial or financial relationships that could be construed as a potential conflict of interest.
